# Spatially resolved sampling of the human oral cavity for metabolic profiling

**DOI:** 10.1016/j.xpro.2021.101002

**Published:** 2021-12-08

**Authors:** Alessio Ciurli, Rico J.E. Derks, Maximilian Liebl, Christine Ammon, Jacques Neefjes, Martin Giera

**Affiliations:** 1 Oncode Institute and Department of Cell and Chemical Biology, Leiden University Medical Center, 2333 Leiden, ZA, the Netherlands; 2 Center for Proteomics and Metabolomics, Leiden University Medical Center, 2333 Leiden, ZA, the Netherlands

**Keywords:** Health Sciences, Clinical Protocol, Metabolism, Metabolomics, Mass Spectrometry

## Abstract

Here, we present a spatially resolved sampling protocol for the oral human cavity aimed at untargeted metabolomics. We describe the spatial collection of salivary biospecimens, their preparation, and subsequent mass-spectrometry-based untargeted metabolomics analysis. Our protocol avoids complex procedures generally required for gland-specific saliva collection. For the human oral cavity, we provide an easy, flexible, and reproducible solution to comprehensively map the highly heterogeneous environment and elucidate the functionality of salivary components.

For complete details on the use and execution of this protocol, please refer to Ciurli et al. (2021).

## Highlights

• Reliable and facile collection of spatially resolved salivary specimens
• Sample preparation of saliva samples for untargeted metabolomics analysis
• Effective workflow for the untargeted metabolomics analysis of saliva

## Graphical abstract

**Figure:**
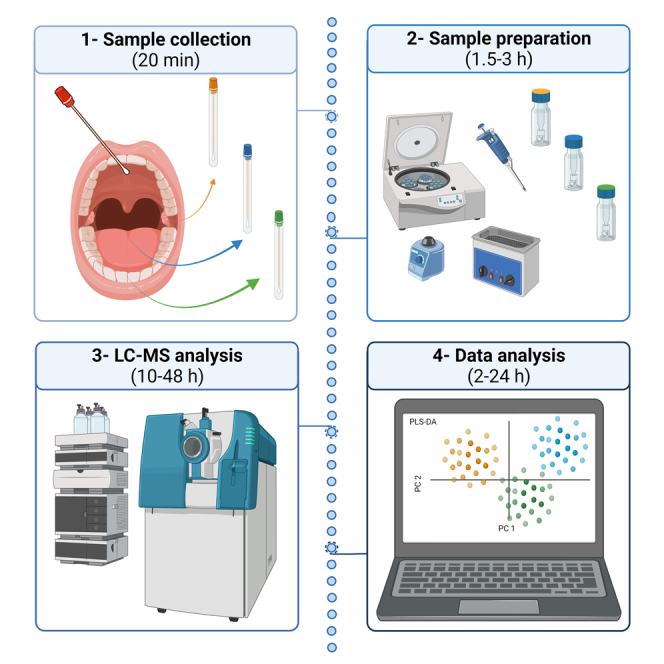

## Before you begin

The presented protocol has been developed for collecting spatially resolved saliva samples. Oral samples are collected from specific, anatomically defined oral locations, namely: cheek, above the tongue and below the tongue. These oral locations are characterized by presence of anatomical borders, teeth and tongue, and the unique combinations of salivary glands. Tongue and palate minor glands are the main source of saliva for above the tongue; submandibular- , sublingual glands, and tongue minor glands are the main glands for below the tongue whereas parotid glands, and cheeks minor glands are the main contributors of saliva for cheeks. The presented protocols can principally be carried out by a third person or the donor him/herself making it ideally suited for (remote)self-sampling. Moreover, the presented spatial sampling procedure should principally be applicable to other specimens such as blister fluid, tears, or wound secretions. Sugi® Eyespear (blunt tip) is the swab selected for a spatially resolved sampling of saliva. The choice has been made after a careful and systemic evaluation, where Sugi® Eyespear has shown a reproducible volume recovery, low background contamination as well as the high coverage and reproducible molecular feature recoveries (Ciurli et al., 2021).

### Sample collection

**Timing: >1 day before day of sampling**

1. Provide all information regarding the precautions and sampling procedure to be followed by the study subjects. Donors are requested to comply with the following precautions prior to sample collection:
  a. Drink 0.5 L of water during the 3 h prior to saliva collection.
  b. Refrain from eating, smoking, drinking (except water), and brushing teeth 1 h before collection.
  c. Rinse the mouth with plain water 10 min before collection.
  d. Talk as little as possible during the 10 min prior samples collection.

Sampling procedures are described in more detail in the step-by-step method details section.

### Sample preparation

**Timing: >2 h**

2. Prepare the following solutions in advance:
  a. 85:15 acetonitrile (ACN):H_2_O (v/v %) for “LC method 1” or 1:99 methanol (MeOH):H_2_O (v/v %) for “LC method 2”.
  b. 10 mM ammonium acetate in H_2_O (eluent A) for “LC method” 1 or 0.1% formic acid in H_2_O (eluent A), and 0.1% formic acid in MeOH (eluent B) for “LC method 2”.
3. Ice cold MeOH: store MeOH at −20°C overnight before sample preparation.
4. Set the temperature of the centrifuge and the autosampler to 4°C.

***Note:*** The amount of solution “a” and “b” and ice cold MeOH to be prepared depends on the number of samples/injections planned.

***Alternatives:*** Other solutions than the one proposed can be used, such as ACN instead of MeOH in solutions a and b for LC method 2. However, chromatographic conditions will require to be optimized consequently.

## Key resources table

**Table:** 

REAGENT or RESOURCE	SOURCE	IDENTIFIER
**Chemicals, peptides, and recombinant proteins**		

Methanol LC-MS grade	Merck	1.06035.2500; CAS: 67-56-1
Water LC-MS grade	Honeywell	14263-2L; CAS: 7732-18-5
Acetonitrile LC-MS grade	Honeywell	34967-2.5L; CAS: 75-05-8
Formic acid additive for LC-MS	Honeywell	56302-10X1ML; CAS: 64-18-6
Ammonium acetate for LC-MS	Honeywell	75594-25G-F; CAS: 631-61-8
ESI positive calibration solution	SCIEX	4463272
ESI negative calibration solution	SCIEX	4463277

**Critical commercial assays**		

Sugi® Eyespear	QUESTALPHA	https://www.questalpha.com/sugi-products/details/product/eyespear
Swab storage tube	Salimetrics	https://salimetrics.com/product/swab-storage-tube-sst-50pk/

**Software and algorithms**		

MS-DIAL (version 4.20)	Tsugawa et al. (2015)	http://prime.psc.riken.jp/compms/msdial/main.html
R (version 4.0.3)	R Core Team	https://cran.r-project.org/

**Deposited data**		

Raw and analyzed data	Ciurli et al., 2021	https://www.ebi.ac.uk/metabolights/MTBLS2905

**Other**		

ACQUITY UPLC BEH HILIC (130Å, 1.7 μm, 2.1 mm × 100 mm)	Waters	186003461
ACQUITY UPLC BEH HILIC VanGuard (130Å, 1.7 μm, 2.1 mm x 5 mm)	Waters	186003980
Synergi Hydro-RP (100 Å, 2.5 μm, 2mm x 100 mm)	Phenomenex	00N-4387-B0-CE
SecurityGuard UHPLC C8 2.1 mm ID columns	Phenomenex	AJ0-8784

## Step-by-step method details

### Sample collection

**Timing: 20 min**

1. Ask the donor to open the mouth.
2. Take a Sugi® Eyespear from an unopened and sterile package.

**CRITICAL:** Using swabs, which have been exposed to air for periods longer than 12 h might result in poor absorption performance.

***Alternatives:*** Sugi® products portfolio is a swab device of different shapes and dimensions which are made of or contain the same medical grade material, therefore we expect the same or similar performance. Although we test only Sugi® Eyespear.

3. Press the Sugi® swab softly onto the surface of the location of interest as described in Table 1.

**Table 1:** Anatomical description and collection tips

Oral location	Anatomical description	How to sample
Above the tongue	This area consists of the center of the body of the tongue, no root and apex.	a. Ask the donor to open the mouth. b. Place the swab on the tongue body. c. Move the swab stick to the side avoiding the front teeth for allowing the mouth to close. d. Ask the donor to close the mouth. e. Ask the donor to gently press the tongue against the palate (Figure 1A).
Below the tongue	This area consists of the floor of the mouth framed by the inferior arch teeth.	a. Ask the donor to open the mouth. b. Placed the swab on the mouth floor. c. Move the swab stick to the side avoiding the front teeth for allowing the mouth to close. d. Ask the donor to close the mouth. e. Ask the donor to press the tongue against the mouth floor (Figure 1B).
Cheek	This area consists of the bottom of the cheeks next to the first molar tooth.	a. Ask the donor to open the mouth. b. Place the swab on the inner cheek in line with the first molar. c. Brush the inner cheek up and down 10 times. d. before Place the swab on the bottom of the cheek (Figure 1C).

For the three oral locations of interest anatomical descriptions and sampling procedure are reported in the table above.

**Figure 1:**
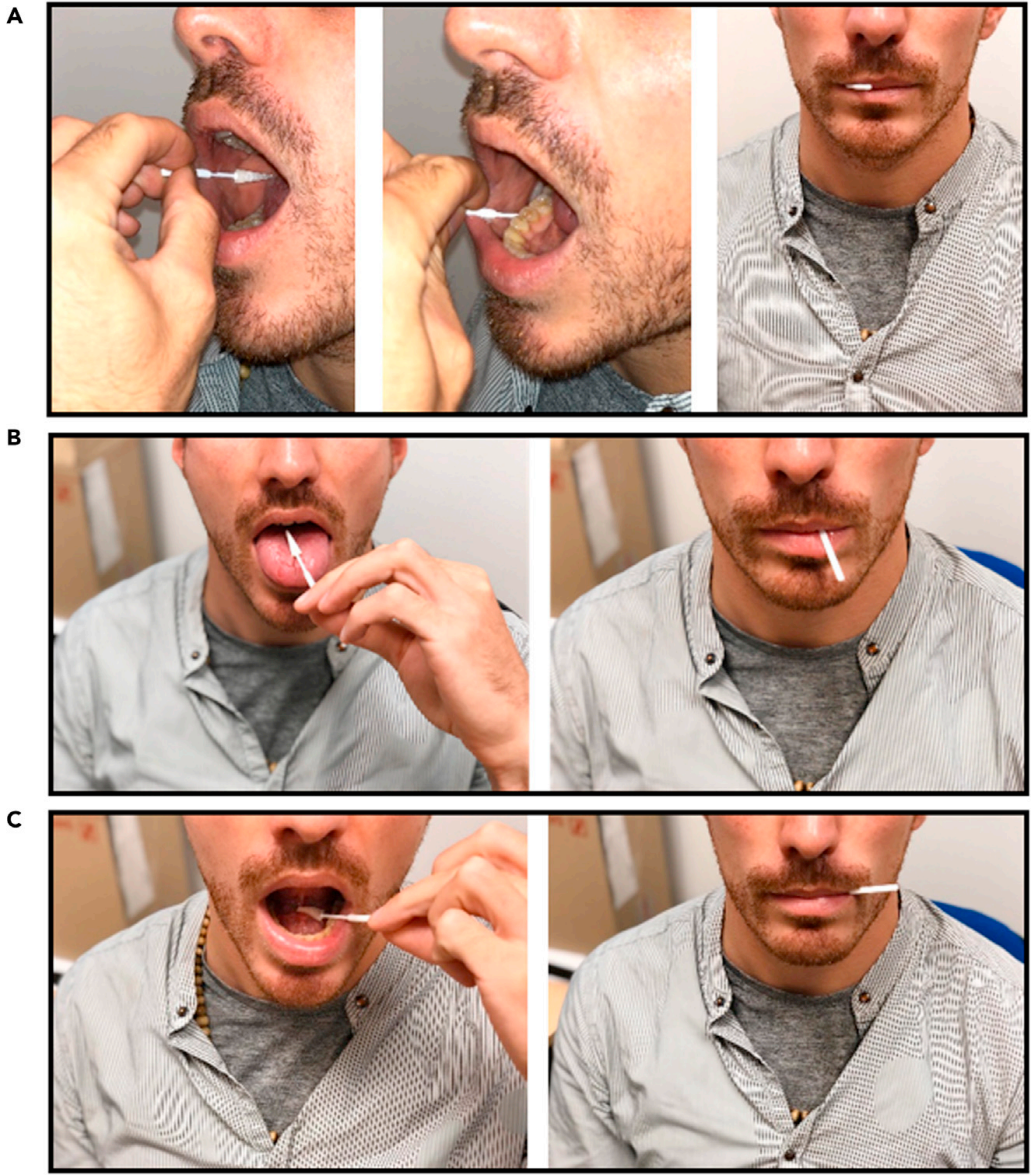
Tips and tricks for sampling (A and B) The three pictures on the top show how to insert the Sugi® absorbent swab into the Salimetrics storage tube (A), whereas the three pictures on the bottom display wrong head positions (marked with a red X) and the correct head position to maintain during sampling procedures (marked with a green check mark) (B).

***Note:*** When placing the swab on the location of interest, be careful to not touch other external or oral surfaces (external surface, lips, teeth, etc.), in order to avoid cross-contamination.

**CRITICAL:** For above- and below- the tongue oral locations, “Table1 - How to sample - step e.” is the essential step to ensure a correct sample collection, absence of contact between the swabs and the oral surfaces of interest will result in low volume or missing collection.

**CRITICAL:** For cheek, “Table1 - How to sample - step c.” is the essential step to ensure a correct sample collection, absence of mechanical stimulation on the inner cheek will result in low volume or missing collection.

4. Ask the donor to close the mouth.

**CRITICAL:** Allowing the donor to keep the mouth open during sample collection might result in a dry mouth and missing sample, especially in the case of multiple collections the mouth must be closed during sample collection.

5. Ask the donor to maintain the head in a neutral position.
6. Wait for 2.5 min.
7. Pull out the swab and place the Sugi® device in a storage tube.

**Figure 2:**
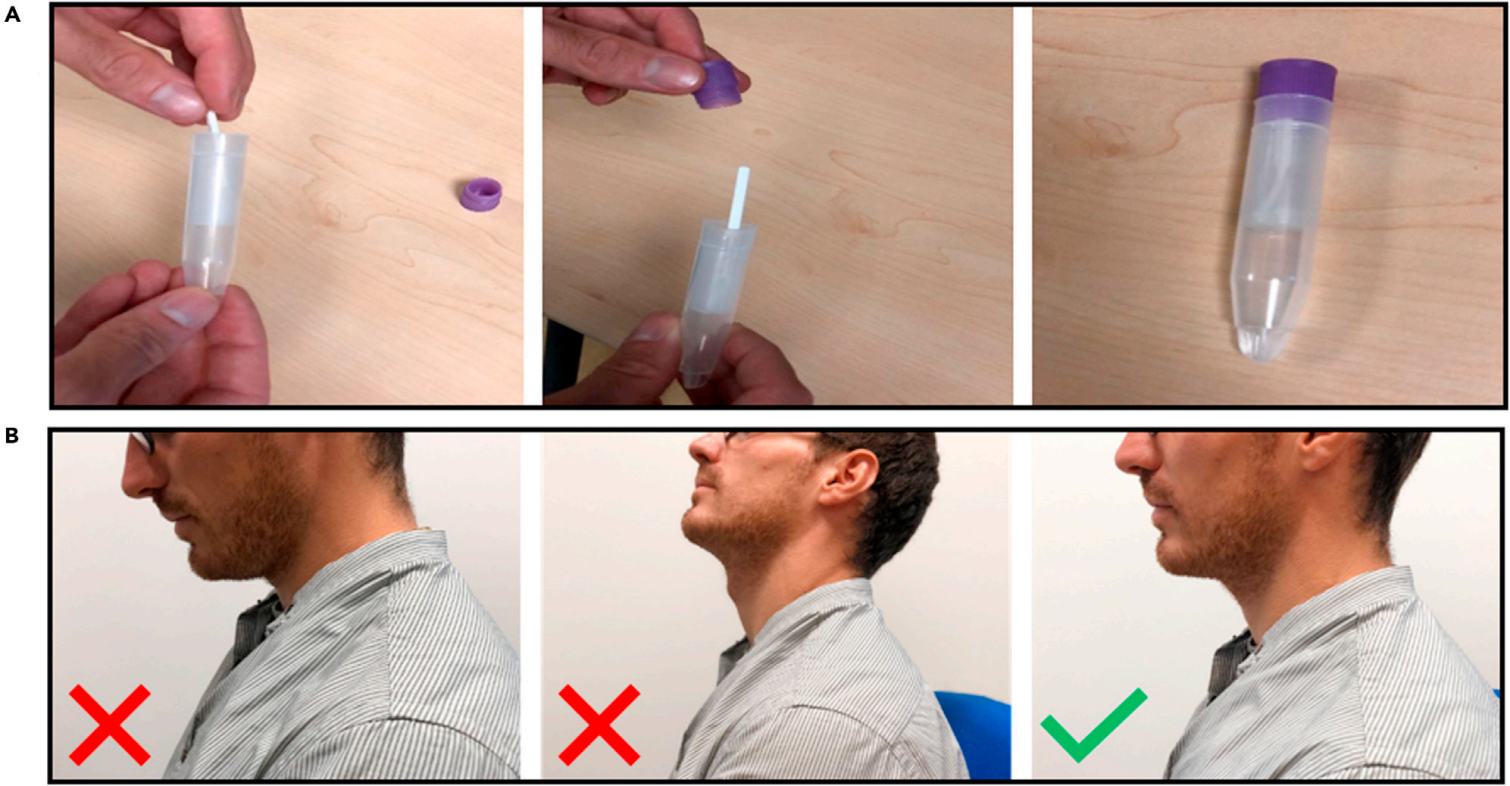
Anatomical descriptions for spatial sampling (A–C) The three pictures on the top are a visual demonstration of how to sample the cheek area (A), the two pictures in the middle show visually how to sample the above the tongue area (B) and the two pictures on the bottom are a visual demonstration of how to sampling the below the tongue area (C).

***Note:*** The swab does not fit completely inside the swab storage tube, but it is possible to squeeze the swab inside using the purple cap (Figure 2A).

**Pause point:** The collected samples can be stored at −20°C for a period shorter than 1 week, for longer periods samples must be stored at −80°C. Several pause points are suggested in this protocol. However, we suggest to limit the freeze-thaw cycle to a single time.

**CRITICAL:** To ensure optimal collection from the cheek area the donor needs to maintain the head in a neutral position (Figure 2B).

### Sample preparation

**Timing: 1.5–3 h**

8. Centrifuge the storage tube at 1500 ×*g* for 15 min at 4°C.

***Note:*** The storage tube has an inner holder tube which allows to centrifuge the saliva out of the swab.

9. Remove the swab from each storage tube after centrifugation.

**Pause point:** The processed samples can be stored at −20°C for a period shorter than one week, for longer periods samples must be stored at −80°C. Several pause points are suggested in this protocol. However, we suggest to limit the freeze-thaw cycle to one single time.

10. After centrifugation, transfer 25 μL from each sample to a 1.5 mL Eppendorf tube for “LC method 1” or 40 μL for “LC method 2”.

***Note:*** The volume to be transferred can be adjusted as needed in order to ensure sufficient material for at least two injections and the aliquot needed to constitute the QC pool samples (step-by-step method details - step 22).

***Optional:*** If the volume collected is sufficient, additional aliquots might be stored as a backup.

11. Control sample. Generate a minimum of six control samples by adding 30 μL of water to a 1.5 mL Eppendorf tube and work up in parallel to your study samples (see below)

***Note:*** The samples of H_2_O are referred as “control” and critical for the statistical analysis.

12. Add ice cold MeOH in a 4:1 (v/v) ratio.
13. Place the samples at −20°C for 20 min.
14. Centrifuge the samples at 18,000 ×*g* for 20 min at 4°C.
15. Transfer 90% of the initial volume of the supernatant into 2 mL glass vials.
16. Dry under a gentle stream of nitrogen.

**Pause point:** The processed samples can be stored at −20°C for a period shorter than 1 week, for longer periods samples must be stored at −80°C. Several pause points are suggested in this protocol. However, we suggest to limit the freeze-thaw cycle to a single time.

17. Re-constitute the dried aliquots with 25 μL of 85:15 ACN:H_2_O (v/v %) for “LC method 1” or with 40 μL of 1:99 MeOH:H_2_O (v/v %) for “LC method 2”.

***Note:*** The volume used to reconstitute the dried aliquots is the same volume as has been transferred after centrifugation (step-by-step method details - step 10).

***Optional:*** Dried aliquots can be re-dissolved in a smaller volume to concentrate the samples.

18. Sonicate for 1 min.
19. Vortex for 5 s.
20. Transfer the prepared solution into a glass vial equipped with a micro-vial insert.
21. Samples are ready for analysis.
22. Prepare a QC pool sample by transferring an aliquot of each reconstituted sample into a glass vial equipped with a micro-vial insert.

***Note:*** Calculate the necessary aliquot size for generating sufficient QCpool sample. Do not add aliquots from the control samples to the QC pool.

23. Prepare a blank sample by filling a glass vial with 85:15 ACN:H_2_O (v/v %) for “LC method 1” or with 1:99 MeOH:H_2_O (v/v %) for “LC method 2”.

***Note:*** Ensure sufficient blank samples for your analysis batch.

### LC-MS/MS analysis

LC method 1: Chromatographic separation is performed using a Nexera X2 UHPLC (Shimadzu) (or comparable) employing an Acquity UPLC BEH HILIC column (130Å, 1.7 μm, 2.1 mm × 100 mm) (Waters). The column is kept at 40°C, and the injection volume is 2 μL. Gradient elution is performed using 10 mM ammonium acetate in H_2_O (eluent A), and ACN (eluent B). The flow rate is 0.4 mL min^-1^. The gradient is as follows: 0–1 min, 95 % B, 9–10 min, 40 % B, 10.5–17.5 min, 95 % B (equilibration).

LC method 2: Chromatographic separation is performed using a Nexera X2 UHPLC (Shimadzu) (or comparable) employing a Synergi Hydro-RP column (100Å, 2.5 μm, 2 mm × 100 mm) (Phenomenex). The column is kept at 40°C, and the injection volume is 2 μL. Gradient elution is performed using 0.1% formic acid in H_2_O (eluent A), and 0.1% formic acid in MeOH (eluent B). The flow rate is 0.4 mL min^−1^. The gradient is as follows: 0–1 min, 95 % B, 9–10 min, 40 % B, 10.5–17.5 min, 95 % B (equilibration).

Mass spectrometry analysis is carried out using a TripleTOF 6600 (Q-TOF instrument, Sciex) (or comparable). The MS instrument is scanning from *m/z* 100 to 1000 for MS1 and from *m/z* 50 to 1000 for MS2 experiments. Measurement conditions are described in Table 2.

**Table 2:** MS settings

Instrument	TripleTOF 6600 (Q-TOF instrument, Sciex)
Source Type	ESI
CUR gas	30
GAS 1	30
GAS 2	25
ISVF	5500 V (−4500 V)
TEM	500°C
Cycle time	0.62 s
DP	80 eV (−80 eV)
CE	10 eV (−10 eV)
Start mass	100 Da
End mass	1000 Da
Accumulation time	50 ms
Acquisition method	IDA
With intensity greater than	100 cps
Maximim number of candidates to monitor per cycle	20
Exclude former target ions	Never
Mass tolerance	50 ppm
CE	30 eV (−30 eV)
CES	15 eV (−15 eV)
Start mass	50 Da
End mass	1000 Da
Accumulation time	26 ms

All parameters required to ensure reproducible MS measurements are reported. When parameters diverge between ESI+ mode and ESI− mode, ESI− parameters are reported in brackets.

### Batch structure

24. For system equilibration the first eight samples of the batch are Injected as follows:
  a. Five equilibration blank samples with a composition equal to the HPLC starting condition.
  b. Two QCpool samples, for further stabilizing the system.
  c. One equilibration blank.

***Note:*** Equilibration samples are for system stabilization only and should be excluded from further data analysis.

25. Subsequently, repeat the following sequence until all the samples are injected:
  a. One QCpool.
  b. Five randomized samples (including prepared control samples (*cf* step-by-step method details - step 11).
  c. One QCpool.
  d. A blank.

***Note:*** The order of samples among all sequences must be randomized in order to avoid batch effects.

***Note:*** The number of QCpool injected corresponds to 20%–25% of the total injections which ensures the analytical consistency of the data.

26. End the batch with the following injections:
  a. A QCpool.
  b. One blank.

***Note:*** Calibration is carried out using commercial (SCIEX) ESI calibration solutions for SCIEX Triple TOF systems. A calibration sample is injected at a flow rate of 50 μL min^−1^ each eight injections after a blank injection. ESI positive calibration and ESI negative calibration solutions are used for positive and negative mode respectively. Calibration samples are not included during batch setup but will automatically be run on Triple TOF 6600 systems.

## Expected outcomes

**Figure 3:**
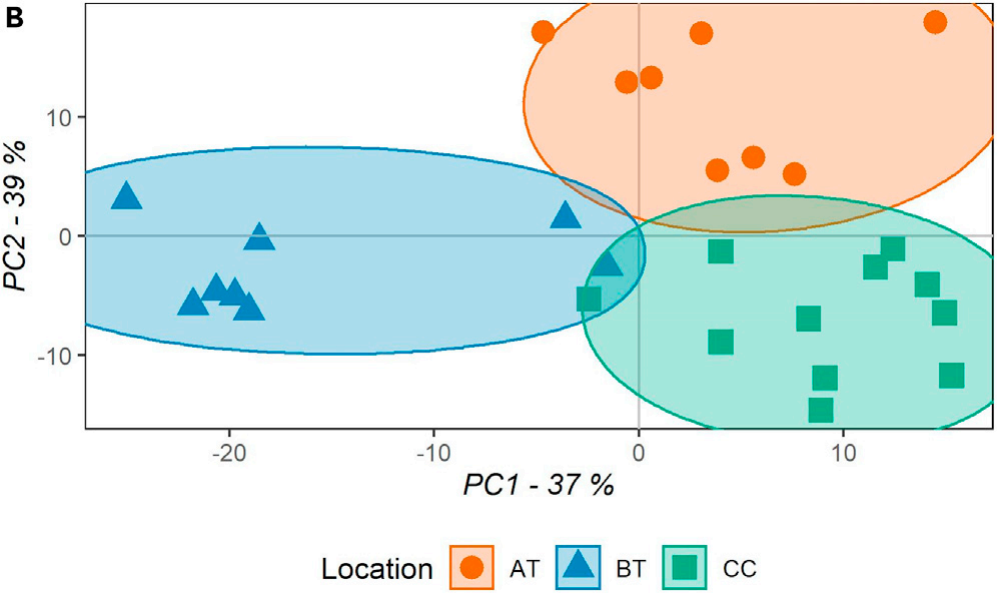
PLSDA score plot The score plot displays the sample distribution for component one (x axis) and two (y axis), on the axis label the explained variance for each component is reported. Samples are colored based on the location of collection (AT = above the tongue, BT = below the tongue, CC = cheeks), as reported in the legend (AT n = 8, BT n = 8 and CC n = 11).

**Figure 4:**
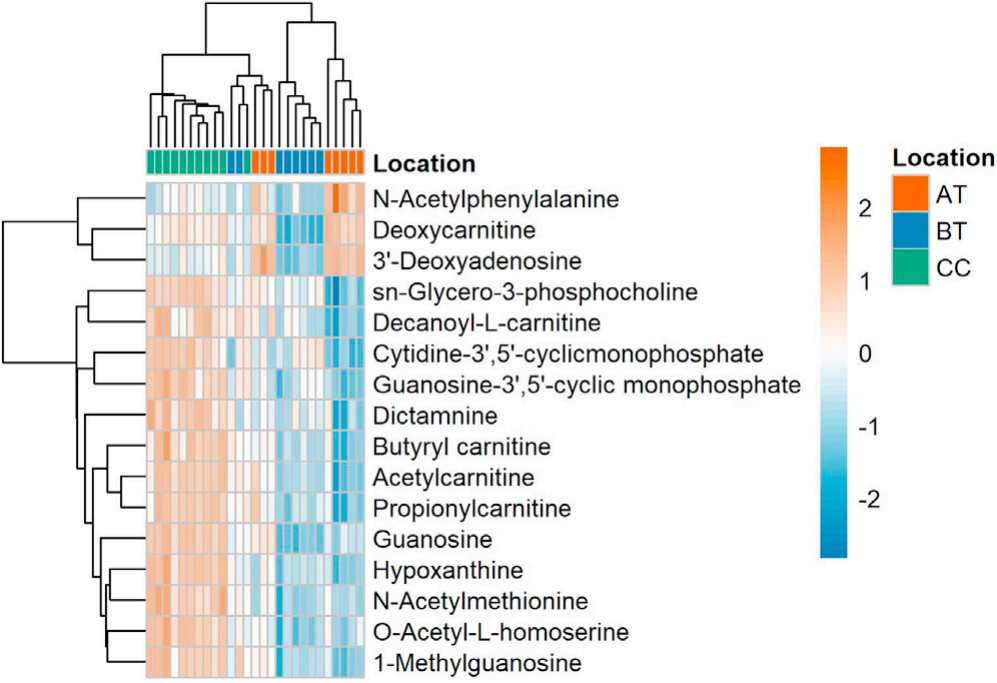
Location characteristic heatmap The heatmap and associated hierarchical cluster of the samples were obtained with the top VIPs (> 1.5) among the identified metabolites. The heatmap was generated using the spatially resolved samples and labelled based on the location of collection (AT = above the tongue, BT = below the tongue and CC = cheeks). Data are centered, UV scaled and log10 transformed (AT n = 8, BT n = 8 and CC n = 11).

**Figure 5:**
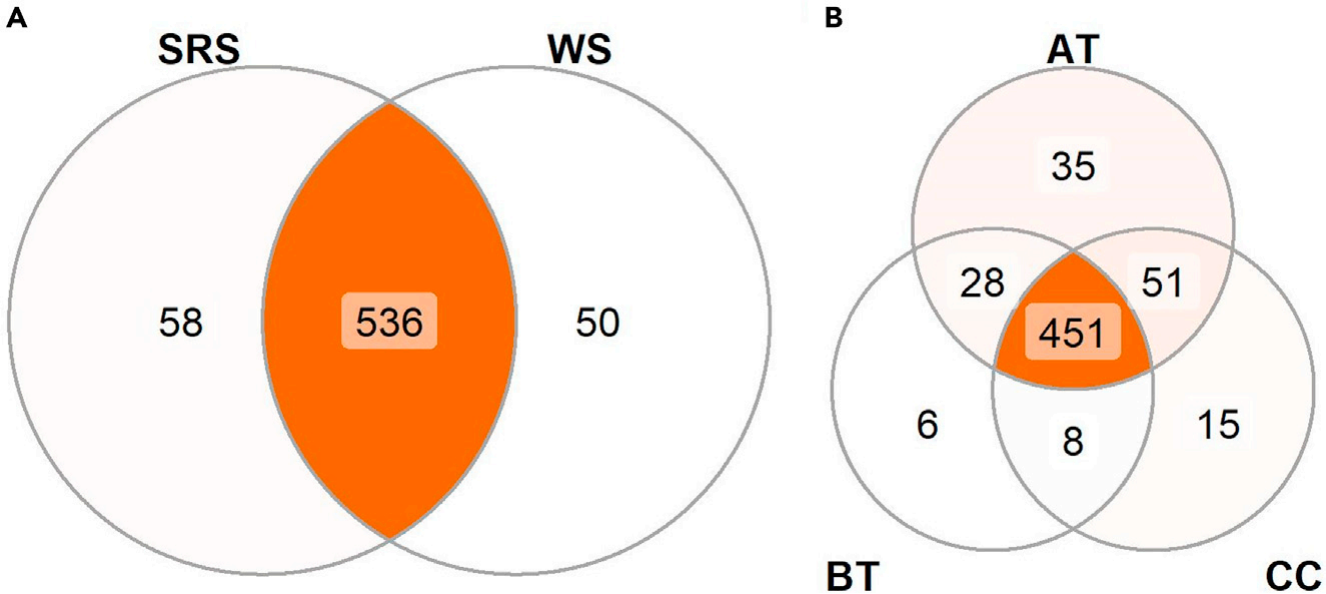
Unique and common analytes The Venn diagrams display the common and unique molecular features per oral location; above the tongue (AT), below the tongue (BT), cheek (CC) (AT n = 8, BT n = 8 and CC n = 11).

A successful sampling should allow a collection of 75–250 μL of saliva per sample, depending on the oral location, time of sampling and intra-subject variability. *Above the tongue* samples display the lowest saliva content ranging from 75 to 150 μL, whereas below the tongue is the location with the highest saliva content between 150–250 μL can be collected. LC-MS analysis of spatially resolved saliva samples should result in distinct clusters based on the oral location of origin (Figure 3), characteristic patterns of analyte distribution among the locations (Figure 4) and detection of unique analytes (Figure 5). For a list with identified metabolites and detected features previously obtained with this protocol, please refer to Ciurli et al. (2021) or metabolights database: MTBLS2905.

## Quantification and statistical analysis

Spectra deconvolution and peak alignment can be performed using MS-DIAL (version 4.20) (Tsugawa et al., 2015). A detailed description of all applied parameters is shown in Table 3. Peaks are considered when present in all replicates of the same sample type. All detected features are validated as follows. (1) In order to remove features which are not sample related, a background threshold (BgT) is computed using an aqueous control sample (worked up LC-MS water sample) (BgT = mean (control_group (peak_area)) + sd (control_group (peak_area)) × 3) (n = 5), consequently every value equal or below the threshold is defined as missing value, (2) features that are not detected in all biological replicates of the reference group are excluded from further analysis. (3) In addition, to evaluate the reproducibility of the measurements, a relative standard deviation (RSD) is calculated for all features detected in the QC pool group (RSD = sd (QCpool_group (peak_area))/mean (QCpool_group (peak_area)) × 100), and consequently a 20 % cut off is applied and any feature characterized by RSD > 20 % is excluded for further analysis.

**Table 3:** MSDIAL settings

MS-DIAL version	4.20
MS1 Data type	Profile
MS2 Data type	Profile
Ion mode	Positive
Target	Metabolomics
Mode	ddMSMS
Retention time begin	0.5 min
Retention time end	10.5 min
MS1 tolerance	0.01 Da
MS2 tolerance	0.025 Da
Mass range begin	100
Mass range end	1000
Maximum charged number	2
Mass slice width	0.1
Smoothing method Linear	Weighted Moving Average
Smoothing level	2
Minimum peak width	8
Minimum peak height	1000
Sigma window value	0.5
MS2Dec amplitude cut off	2
Exclude after precursor	True
Keep isotope until	0.5
Keep original precursor isotopes	False
MSP file	MSMS-Public-Pos-VS15
Accurate mass tolerance (MS1)	0.01 Da
Accurate mass tolerance (MS2)	0.05 Da
Identification score cut off (%)	75
Using retention time for scoring	False
Using retention time for filtering	False
Retention time tolerance	0.1 min
Accurate mass tolerance	0.01 Da
Relative abundance cut off	0
Top candidate report	True
**Adduct ions**	[M+H]^+^, [M+NH_4_]^+^, [M+Na]^+^, [M+K]^+^, [M+ACN+H]^+^
Retention time tolerance	0.05 min
MS1 tolerance	0.015 Da
Retention time factor	0
MS1 factor	0.5
Peak count filter	0
N % detected in at least one group	100
Gap filling by compulsion	False
Tracking of isotopic labels	False
Ion mobility data	False

## Limitations

The presented protocol describes a facile, versatile and reproducible sample collection method for untargeted metabolic profiling of saliva in a spatially resolved fashion. If the protocol is being used for other bodily fluids, e.g., tears, blisters, or wound fluid a brief validation of the protocol for these purposes should be considered. Moreover, we have not developed the protocol for quantitative analysis. In other words, if the protocol is being applied for the targeted analysis of specific metabolites a dedicated validation procedure must be performed. Also, when the protocol is used for sampling specific disease areas, data normalization should be considered. This can be achieved by different approaches for example total area normalization as well as normalization based on collected area or volume. Ultimately, each study has its own limitations and requirements and the suitability of our protocol for specific purposes should be investigated on an individual basis.

## Troubleshooting

### Problem 1

Missing samples.

### Potential solution 1

In order to avoid missing samples, we suggest to centrifuge each storage tube before freezing (*cf* step-by-step method details - step 8), with the aim of knowing ahead the number of missing samples by visual inspection. Subsequently, sample collection can be repeated with the same subjects whose samples are missing, when possible, or recruiting new subjects up to the desired number of samples.

### Problem 2

Low volume of saliva collected.

### Potential solution 2

When the minimum volume of saliva do not reach the necessary volume for the LC-MS/MS analysis (HILIC = 25 μL, RP = 40 μL), the volume collected can be increased by applying mechanical stimulation through gentle brushing of the oral location of interest prior the absorption time, increasing the absorption time (step-by-step method details - step 3) or both.

### Problem 3

Saliva samples contaminated with blood.

### Potential solution 3

The presence of blood profoundly alters the profile of the biological sample collected and constitutes a serious confounding factor during data analysis. Make sure to exclude subjects with gum bleeding or poor oral hygiene by using strict exclusion and inclusion criteria.

### Problem 4

Altered saliva profile.

### Potential solution 4

Exogenous compounds, such as foods and drugs, might have a large influence on the metabolomic profile of saliva. In order to identify the source of changes, we suggest the use of a questionnaire to annotate food and drugs intake of the study subjects or, alternatively, to prevent alteration of the metabolomic profile, drugs intake might be added into the exclusion criteria whereas stricter sampling precautions might be implemented to avoid alteration caused by food intake, e.g., 12 h overnight fasting (before you begin - step 1).

### Problem 5

High variance in results.

### Potential solution 5

In our experience, the saliva composition is largely influenced by the circadian rhythm. As a result, sampling donors at the same time of the day can effectively reduce the variance of the analysis results.

## Resource availability

### Lead contact

Further information and requests for resources and reagents should be directed to and will be fulfilled by the lead contact, Martin Giera (m.a.giera@lumc.nl).

### Materials availability

This study did not generate new unique reagents.

## Data Availability

This study did not generate any dataset or original code; all codes used are mentioned in the statistical analysis section.
